# HIV Awareness and Risk Behavior among Pregnant Women in Mateete, Uganda (2010)

**DOI:** 10.5402/2011/709784

**Published:** 2011-11-30

**Authors:** Josefin Sandqvist, Johanna Wahlberg, Elly Muhumuza, Rune Andersson

**Affiliations:** ^1^Institute of Biomedicin, The Sahlgrenska Academy, University of Gothenburg, Guldhedsgatan 10A, P.O. Box 7193, SE-40234 Gothenburg, Sweden; ^2^Bamu Hospital, Mateete, Uganda

## Abstract

*Background*. The aim of the study was to evaluate current knowledge, risk behavior, and attitudes among pregnant women in Mateete, Uganda. *Methods*. We collected 100 questionnaires and performed 20 interviews among women who attended antenatal care. *Findings*. All the women had heard about HIV/AIDS, and 92% were aware of mother-to-child transmission. The women overestimated the risk of achieving the virus since 45% believed in transmission by mosquitoes and 44% by kissing. Many pointed out that married women as a group were infected more often because of unfaithful partners who refused to use condoms during sex. *Conclusion*. The women were well aware of the routes of HIV transmission. Schools and governmental campaigns have played an important role in educating people about the disease but there is still a great need to reach out to people in rural areas with both health care and correct information.

## 1. Introduction

In 2009 it was estimated that 3.4 million people are living with HIV in the world [1]. Only 33% of HIV-positive women received antiretroviral drugs to reduce the risk of mother to child transmission in 2007 [2]. 

The Republic of Uganda is located in East Africa where HIV was first discovered in the 1980s. It spread rapidly, and by the early 1990s the average national HIV prevalence in Uganda was 18% in rural areas and 25%–30% in major urban areas. Through political commitment and openness about the epidemic, the prevalence and incidence declined during the following decade to just 6-7%. The first National AIDS Control Program, Uganda AIDS Commission (UAC), was established in 1992 [1]. The aim was to educate the Ugandan people about how to prevent HIV infection using the Abstinence, Be faithful, correct/consistent Condom use (ABC) strategy [3]. 

The transmission of HIV from mother to child is the second most common route of transmission of HIV in Uganda [4]. The risk of HIV transmission from mother to child is approximately 45% if no safety measures have been taken. With early HIV testing, special precautions during delivery, prophylactic treatment, and shortened breast feeding, the risk can be reduced to only a few percent [5]. 

Bamu Hospital is a private hospital situated in the village of Mateete in the southern part of Uganda [6]. It is situated in the Central Region which has the highest prevalence of HIV in the country, 8.5%. Mateete has a population of about 5000 people and most of them rely on farming [4]. 

Bamu Hospital fulfills the government of Uganda's generally accepted standards regarding prevention of mother to child transmission by offering pre- and post-HIV test counseling for pregnant women, by counseling HIV-positive women on infant feeding practices, by providing prophylactic HAART (Highly Active Antiretroviral Therapy) to HIV-positive women during pregnancy and delivery, by giving zidovudine syrup to the newborns, and by providing family planning [4]. 

To increase the number of healthily born babies in the country, a campaign called “Healthy Baby” was run by Marie Stopes International and financed by the Global Partnership on Output-Based Aid (GP OBA) together with the Federal Republic of Germany through KFW Entwicklungsbank. “Healthy Baby” has been carried out in Uganda since 2008. The idea is to enable pregnant women to attend antenatal care every month throughout their pregnancy, to deliver at a health facility, and to receive postnatal care for less than one euro [7]. All women in our study were enrolled in this campaign.

## 2. Aim of the Survey

The aim of this study was to investigate the knowledge and the attitudes towards HIV and AIDS among pregnant women in Mateete, Uganda. We compared our results with similar studies made in Tanzania, China, Kazakhstan, and Hong Kong.

## 3. Method and Participants

The study was conducted between November 8 and December 5, 2010 at Bamu Hospital, Uganda. To improve the reliability and accuracy we collected 100 questionnaires and conducted 20 interviews. The study took place over a period of four weeks to make sure that we did not meet the same women more than once, since they came monthly according to the Healthy Baby campaign [7]. The women were in various stages of their pregnancies and they answered our questions after they had seen the midwife.

All the women were informed that their participation was voluntary and that they were free not to answer the questions they found too personal. They were also informed that we would store the information confidentially and that they would not be identified. After this they gave oral consent.

In the first week we handed out questionnaires to all women who came for antenatal care. In the second and third week we conducted two random interviews a day, the first woman who came in the morning and the first who came in after lunch. In the last weeks we finished collecting questionnaires. Since the majority of the women could not read in English or their maternal language we relied on interpreters. All women but one needed an interpreter. The interpreters were nurse students who spoke excellent English as well as a variety of different maternal languages which made it possible for us to enroll almost all women. During our study we worked with nine students. We introduced them to our questionnaire and instructed them how to interpret with standardized objectivity. We were always available to clarify questions.

The questionnaire was originally constructed by Professor Glen Mola at Port Moresby General Hospital, Papua New Guinea [8]. We modified it to make it suitable for our study. The questionnaire contained 43 questions divided in four categories: general questions, questions about HIV/AIDS, private life, and HIV/AIDS and pregnancy. The questionnaire starts out with questions about age, education, profession, living conditions, and the family situation. The second part consists of questions asking if they have heard about HIV/AIDS, when and how they first heard about it, knowledge about the difference between HIV and AIDS, how they think it is spread, if there is a way to protect oneself, and if they know of any treatment. Knowledge about symptoms of the disease and attitudes towards special groups is also investigated, as well as the participant's estimate of the number of persons infected with HIV in their area. Thereafter it follows questions about the number of sexual partners, age at sexual debut, as well as HIV risk behavior and HIV-status. The last part asks questions about if the women were informed about HIV and if they were offered an HIV test when they got pregnant. The women who tested positive were then asked to answer six more questions about antiretroviral treatment, Caesarian section, breastfeeding and HIV test for their coming child.

The interviews were semistructured, and we relied upon interpreters, the same nurse students we used for the questionnaires since none of the women spoke English. The women were asked questions concerning four different areas: background, general knowledge of HIV/AIDS, personal attitudes, sexuality, and additional information. We tried not to ask leading questions.

### 3.1. Participants

A total of 120 women participated in our study, 100 answered our questionnaire, and 20 were interviewed. During the time that we performed our study, 158 women attended antenatal care at Bamu. The women who did not answer were either too sick to participate or excluded because our interpreters were unavailable at the moment. We have built our statistics on our questionnaire while the interviews have been used to enlighten certain topics of special interest. The results of the interviews are presented as comments continuously in the text.

## 4. Results

The ages of the 100 women in the questionnaire group ranged between 15 and 46, the mean age was 21.3, and the median age was 22. The ages in the interview group ranged between 17 and 32 years; the mean age was 24.8 and the median 24.

The number of children still alive in the questionnaire group ranged from 0 to 7. The mean and the median were 1. Four women in this group did not answer if they had children or not, therefore these women were excluded when we presented our data over the number of children. The number of children still alive in the interview group ranged between 0 and 6, the mean was 3.1, and the median was 2.

### 4.1. General Information

Out of the women who answered our questionnaire, 90% were married. Eighty-four percent reported that their children had the same father, and 16% reported that their children had different fathers. Seventy-five percent lived in a house, 12% lived in an apartment, 10% lived with relatives, and 3% had no stable place to live. Eighty-eight percent answered that they had enough food and 12% said that they did not. Four percent were employed; 79% were self-employed; 27% were not employed.

Eight percent in the questionnaire group had never been to school; 41% attended primary school but did not finish; 33% finished primary school; 16% attended secondary school; 1% attended high school; and 1% attended university. Most women we interviewed had attended school, but few had been able to finish primary school. HIV education was common in school, but sexual education was less common.

### 4.2. Questions about HIV/AIDS

All the women had heard about HIV/AIDS.

**Figure 1:**
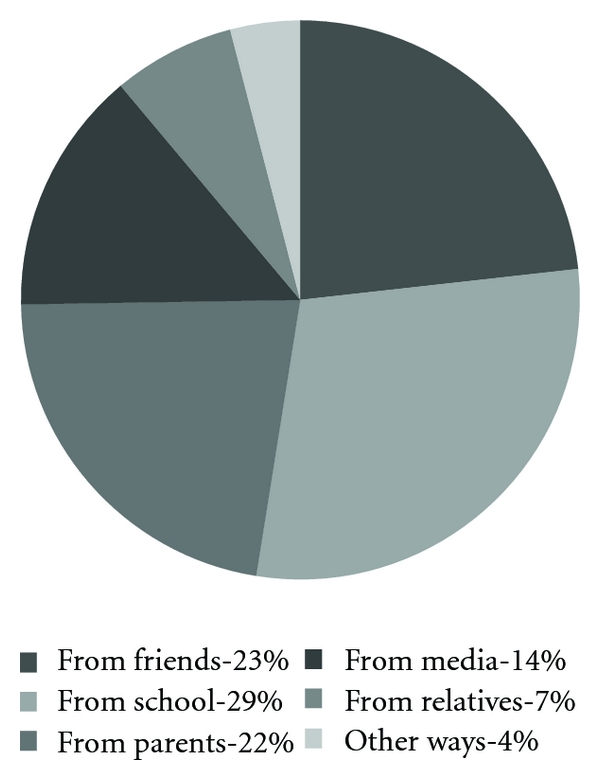
From where did you first hear about HIV/AIDS? (*n* = 99).

Figure 1 shows from where the women first heard about HIV/AIDS.

**Figure 2:**
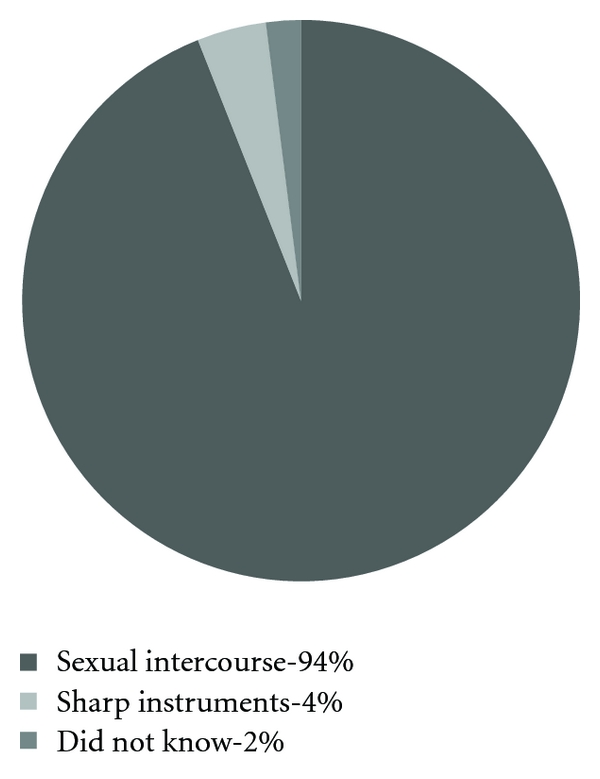
Which is the main route that HIV is spread through? (*n* = 100).

Figure 2 shows which main route the women thought that HIV is spread through.

Eighty-five percent knew there was a way to protect yourself; 5% did not think there was a way to protect; 8% did not know; 2% did not answer. The ways to protect yourself that were mentioned were condom use, to abstain from sex, and by being faithful to your partner.

When asked if any special group is more often infected than others, 25% answered youth, 22% married people, 7% prostitutes, 6% people with tuberculosis, 3% old people, 3% men, 1% unmarried, 1% divorced, 1% women, 1% orphans, 1% people with malaria, and 1% bar staff. 16% did not think any special group was more often infected, and 11% did not know. Many women we interviewed also said that married people are more often infected. They explained that husbands are often unfaithful and that their wives are unable to protect themselves because it is a man's right to have sex with his wife without using a condom. Most women we interviewed would not leave their husband even if he was HIV positive.

Fifty-five percent thought that you can see if someone has HIV by looking at the person, 36% did not think so, and 9% did not answer. When we asked them to be specific, they answered the same as they did when they were asked to specify symptoms of AIDS. Many mentioned herpes zoster, Kaposi sarcoma, skin rashes, weight loss, on and off fever, anemia, depression, lymph nodes, oral candidiasis, wounds in the mouth, loss of appetite, diarrhea, vomiting, spots on skin, and bruises.

When asked how HIV is spread the women answered as shown in Table 1.

**Figure 3:**
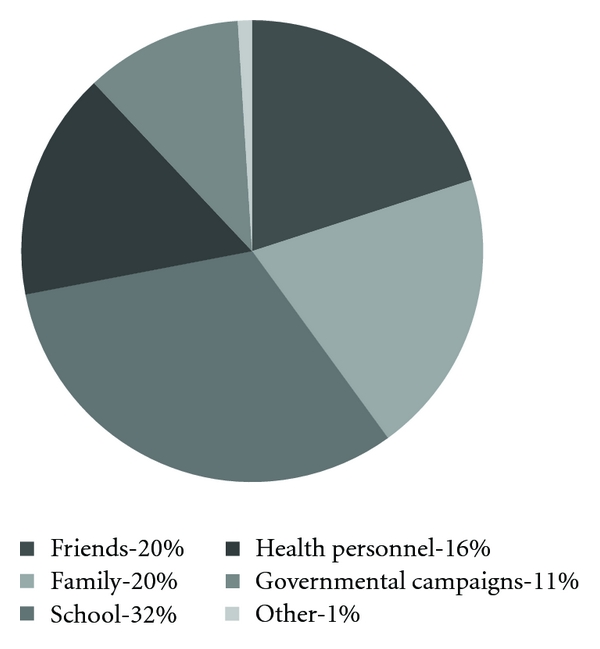
Where did you get the most information about HIV? (*n* = 100).

Where the pregnant women in Mateete got most information about HIV/AIDS is shown in Figure 3. When asked if they think they have got sufficient information about HIV/AIDS to protect themselves and their children, 69% answered yes, 28% answered no, and 3% did not know. Among those who wished to know more, 68% said they wanted to be informed by health personnel, 14% said school, 14% said relatives, and 4% answered written information. 

**Figure 4:**
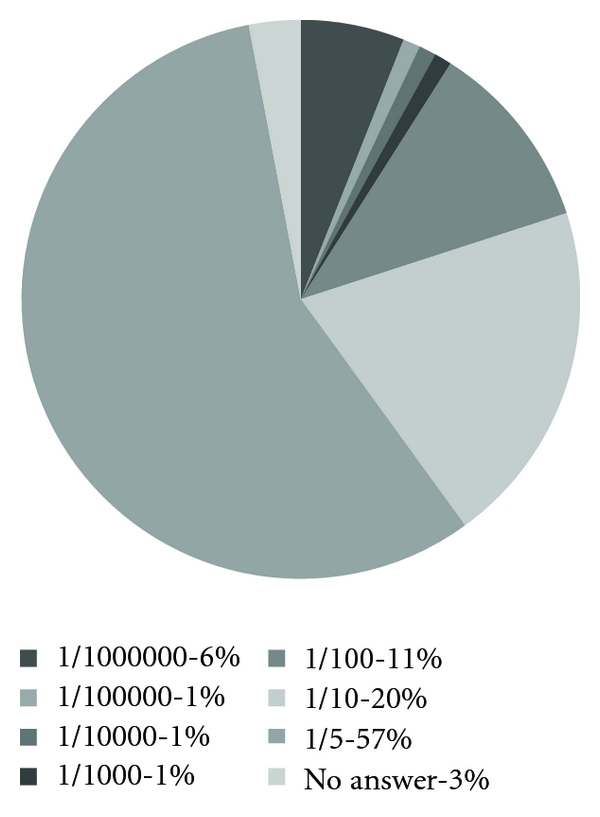
How many people are infected in the area where you live? (*n* = 100).

How many people the pregnant women thought were infected with HIV/AIDS in the area where they live is shown in Figure 4. 

Many of the women we interviewed thought that the number of HIV-infected persons in the area where they live was above 50%. They believed this because that was the number of people who looked sick in one way or another, possibly because they were coughing, had diarrhea, skin rashes, or looked weak. 

Fifteen percent of the women thought that there was a medical cure for HIV/AIDS whereas 81% thought there was not and 4% did not know. When we asked them if there were any other ways to cure HIV/AIDS, 16% answered that there were, 59% said there were not, 22% did not know, and 3% did not answer. When asked to specify what the cure would be they mentioned herbs and that the TASO organization (The AIDS Support Organization Uganda) sometimes can cure. On the question of whether there was a way to slow down the progression of the disease 83% answered yes, 9% answered no, and 8% did not know.

### 4.3. Private Life

**Figure 5:**
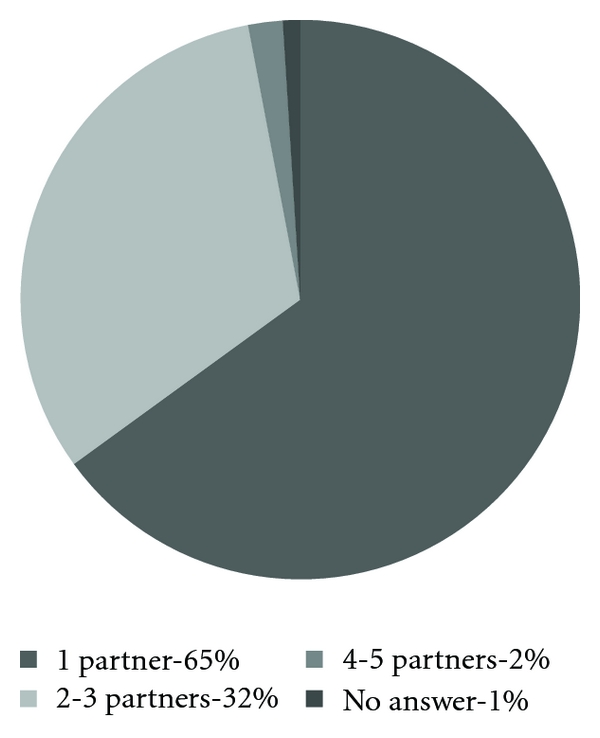
The number of lifetime sexual partners. (*n* = 100).

The number of lifetime sexual partners varied from 1 to 5, and how it varies is shown in Figure 5. 

Most of the women that we interviewed thought that people in general have more than one sexual partner during a lifetime, but when asked specifically about themselves, they had only had one. They also believed that men have more sexual partners than women. 

Two percent of the women were under 10 years old when they had their first sexual contact, 19% were 10–15 years, 72% were 16–20 years, 5% were 21–25 years, and 2% did not answer. 

When the women met their current partners 48% asked about previous partners, 46% did not ask, 4% did not know, and 2% did not answer. Forty-six percent asked their partner to take an HIV test, 47% did not, 3% did not know, and 4% did not answer this question. Twenty-nine percent asked their partner to test for other sexually transmitted diseases, 63% did not ask for this, 3% did not know, and 5% did not answer. 

The number of women who answered that they had been tested for HIV was 81%. Of the 19% that had not been tested 74% wanted to take an HIV test, 21% did not want to, and 5% did not know. 

**Figure 6:**
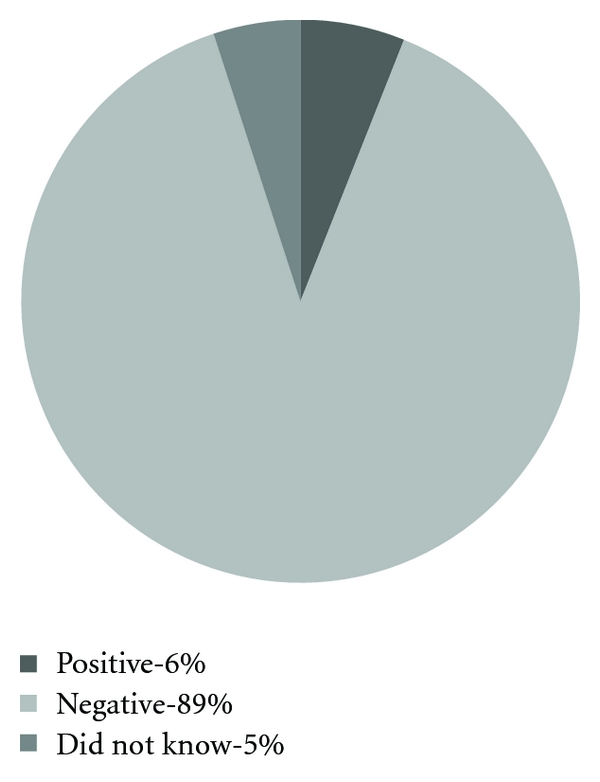
HIV status among the women who had been tested. (*n* = 100).

HIV status among those women who answered that they had been tested is shown in Figure 6. The reason why the women who had been tested for HIV took the test was in 48% pregnancy, 33% before marriage, 7% because of their worry that their current partner was infected, 7% because of their worry that their previous partner was infected, 1% because their partner was infected, 1% because of illness, and 1% did not answer. Among those who knew that their partners had been tested 89% knew the result to be negative, 9% did not know the result, and 2% knew it to be positive. 10% of the women had tested their children for HIV, 57% had not, 28% expected their first child, and 5% did not want to answer. None of the children tested positive. 

**Figure 7:**
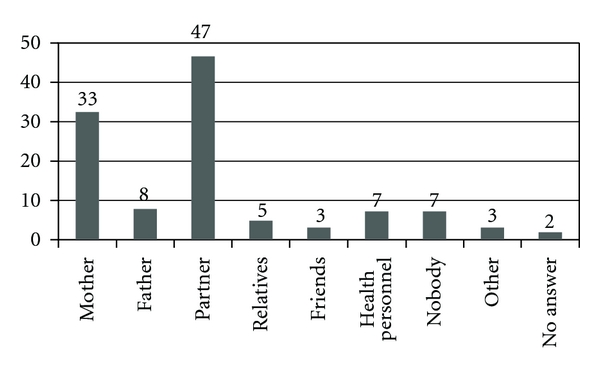
Who the women would tell if they were tested HIV-positive (the women could choose several). (*n* = 100).

When asked who they would tell if they were found to be HIV positive the women could choose from several alternatives. The result is shown in Figure 7. Almost all of the women that we interviewed thought that their families would support and treat them the same if they were diagnosed with HIV. A minority thought that they would be excluded from their families. About half of the women answered that they would stay with a friend even if he/she was HIV positive and would treat him/her the same.

### 4.4. HIV/AIDS and Pregnancy

Half of the women said that they got information about HIV/AIDS when they got pregnant, 46% did not get any information, and 4% did not answer. 71% were offered an HIV-test when they got pregnant, 27% were not, and 2% did not answer.

## 5. Discussion 

### 5.1. Knowledge

We were happy to find that 100% of the women that either answered our questionnaire or were interviewed had heard about HIV. This corresponds to the findings in Ilembula, Tanzania [9]. We were also glad that 99% knew that HIV can be transmitted through sexual intercourse without using a condom and that 94% knew that this is the main route. This is comparable to the studies performed in Illembula, Tanzania [9], Aksu, China [10], and Semey, Kazakhstan [11]. Since the women answered our questions after they had met health care personnel who were supposed to inform about HIV/AIDS and since many of them already had been engaged in antenatal care before, we conclude that antenatal care improved their knowledge about HIV/AIDS. This is the general standard and a part of the government of Uganda's strategy to prevent HIV/AIDS transmission from mother to child [4]. 

Since as much as 92% of the women had been to school and as we acknowledged in the interviews that the schools around Mateete teach their students about HIV/AIDS, it is not surprising that most women had first heard about HIV in school. 

School was the place where the women learnt the most about HIV/AIDS and is, we believe, an efficient way of spreading information about HIV/AIDS. This motivates targeted information and should be kept in mind when setting up future informational campaigns. One thing that is surprising is that even though most women in our study had been to school, few knew how to read and write well enough to fill in the questionnaire. This may be explained by the fact that many of them did not finish primary school. Many had first heard about HIV/AIDS from either friends or parents which tells us that these also are important sources of information in rural areas. This corresponds to the findings among women in Aksu [10] and Semey [11]. 

Despite the widespread knowledge about the virus, there are still many misconceptions. Many women had difficulties distinguishing the difference between HIV and AIDS. This corresponds well with the study made in Semey [11]. Fifty-five percent thinks that you can tell if someone has HIV/AIDS by looking at the person. Other misconceptions concerned how the women believed that HIV could be spread. In addition to sexual intercourse as the main route many knew that breastfeeding, sharing needles, and mother-to-child transmission are important when it comes to spreading HIV. Many women also believed that both kissing and mosquitoes were possible routes, which indicates that they exaggerate the risk of achieving the virus. This misconception about mosquitoes is in agreement with the Semey study [11]. 

Most of the women were able to exclude daily domestic contacts such as eating and drinking from the same plates or cups, hugging, shaking hands, and changing clothes with an HIV-positive person as a possible route of spreading the virus. This is even better than the result from the Illembula [9] study which is reassuring. The women in our study were not able to exclude the risk of kissing as a possible route, which the women in the Ilembula [9] and the Semey [11] studies could do.

During our interviews we found out that many women thought that 50% or more in their area were infected. When we analyzed the data from our questionnaires regarding this question, we realized that the alternatives that were available only ranged between 1/1000000 and 1/5. Many had ticked in 1/5 and written a higher percentage next to the box. This shows that they also overestimate the number of infected persons in their area and that many live with a greater fear of the virus than necessary. 

Another reassuring finding is that as many as 85% know that there are ways to protect themselves from HIV. They specify by mentioning abstaining from sex, being faithful, and using condoms which literally comes from the governmental campaign ABC [3]. The women who did not answer that there is a way to protect were often married. Married people were also pointed out as one group that are more often infected with HIV/AIDS which puzzled us before we got their explanations in the interviews. The women then told us that this is simply because married women cannot protect themselves if their husbands are unfaithful or have more than one wife. 

Most women believed that they had got sufficient information about how to protect themselves and their children. Among those who did not think so, the majority wished to be informed by health personnel. About half of the women said that they got information about HIV when they got pregnant, and three quarters of them said that they had been offered an HIV-test. According to the Healthy Baby campaign [7] all women who attended antenatal care should be tested for HIV for free. Therefore all women had been tested, but they did not know this. This implies that health personnel could put more effort into communicating about HIV. 

Compared to the women in Semey [11] most of the women in our study were well aware of the fact that there is no cure for HIV. 

About just as many women were also aware that there is a way to slow down the progression of the disease. Some also believed that there are other ways to treat HIV than medicine, like herbs. This misconception is of course important to educate people about, so that they are more likely to seek medical care instead of traditional cures.

### 5.2. Risk Behavior

Most women reported that they had only had one sexual partner. When asked about people in general in Uganda many of the women that we interviewed said that it is common to have more than one, but that they had only had one. Because we relied on interpreters and because we were often around to answer questions they might have underreported the number of sexual partners that they had had. 

Eighty-one percent of the women said that they had been tested for HIV and the main two reasons why were either pregnancy or that they were getting married. This is a very high rate compared to the Illembula study [9] where only 13% had been tested. The Healthy Baby campaign might be a reason that the number is this high. The fact that marriage is a common reason why the women have been tested, and that many ask their partners to be tested indicates that they are well aware of the HIV situation and that they try to be cautious. 

Less than half of the women would tell their partner if they became HIV positive. This is a low number compared to the Illembula study where 85% would tell their partners [9]. Many women also mentioned that they would tell their mothers. In both our study and the Illembula study [9] the women were able to choose several alternatives. Since few did so in our study, we have reason to believe that they did not understand that they had this opportunity. 

Even though the knowledge among women in our study was satisfying, and the possibility of getting treatment was good, this could not speak for Uganda in general. According to Uganda Service Provision Assessment Survey from 2007 [4] these services are not accessible for everyone. The Healthy Baby campaign [7] was an attempt to reach out to a bigger group, and we believe that this is an important step in the right direction. Every effort should be made to ensure that antenatal care is affordable and accessible to every pregnant woman.

## 6. Conclusions

- All of the women in our study had heard about HIV, and almost all of them knew how you acquire the virus. This shows that the governmental campaigns have been successful.
- The women overstate the risk of achieving the virus and the number of people that are infected. This leads to stigmatization and makes people live with greater fear than necessary. Further education is still important to spread correct information.
- Even though every pregnant woman was tested for HIV many did not know their status and many did not know how to protect their child(ren) and themselves. This could be improved by more information from the health personnel.
- Less than half of the women would tell their partner if diagnosed HIV positive.
- Many reported an increased risk for HIV among married women.

## Figures and Tables

**Table 1:** How can HIV be spread from one person to another? (*n* = 100).

	Yes	No	Do not know	Did not answer
Eating or drinking from the same plates and cups?	7%	82%	10%	1%
Shaking hands, hugging, living in the same house?	5%	88%	4%	3%
Changing clothes with someone who has HIV/AIDS?	6%	87%	6%	1%
Kissing?	44%	42%	14%	—
Sexual intercourse without condom?	99%	1%	—	—
Sexual intercourse with condom?	18%	72%	9%	1%
Sharing needles while injecting drugs?	94%	3%	2%	1%
Breastfeeding?	83%	10%	7%	—
From mother to child during pregnancy or delivery?	92%	4%	4%	—
By mosquitoes?	45%	38%	16%	1%
